# Statins affect *ETS1*-overexpressing triple-negative breast cancer cells by restoring *DUSP4* deficiency

**DOI:** 10.1038/srep33035

**Published:** 2016-09-08

**Authors:** Hae Hyun Jung, Soo-Hyeon Lee, Ji-Yeon Kim, Jin Seok Ahn, Yeon Hee Park, Young-Hyuck Im

**Affiliations:** 1 Department of Health Sciences and Technology, SAIHST, Sungkyunkwan University, Seoul 06351, Korea; 2 Division of Hematology-Oncology, Department of Medicine, Samsung Medical Center, Sungkyunkwan University School of Medicine, Seoul 06351, Korea; 3 Biomedical Research Institute, Samsung Medical Center, Seoul 06351, Korea.

## Abstract

We investigated the molecular mechanisms underlying statin-induced growth suppression of triple-negative breast cancer (TNBC) that overexpress the transcription factor ets proto-oncogene 1(ets-1) and downregulate dual specific protein phosphatase 4(dusp4) expression. We examined the gene expression of BC cell lines using the nCounter expression assay, MTT viability assay, cell proliferation assay and Western blot to evaluate the effects of simvastatin. Finally, we performed cell viability testing in TNBC cell line-transfected *DUSP4*. We demonstrated that *ETS1* mRNA and protein were overexpressed in TNBC cells compared with other BC cell lines (*P* = <0.001) and *DUSP4* mRNA was downregulated (*P* = <0.001). MTT viability assay showed that simvastatin had significant antitumor activity (*P* = 0.002 in 0.1 μM). In addition, simvastatin could restore dusp4 deficiency and suppress ets-1 expression in TNBC. Lastly, we found that si-DUSP4 RNA transfection overcame the antitumor activity of statins. MAPK pathway inhibitor, U0126 and PI3KCA inhibitor LY294002 also decreased levels of ets-1, phosphor-ERK and phosphor-AKT on Western blot assay. Accordingly, our study indicates that simvastatin potentially affects the activity of transcriptional factors such as ets-1 and dusp4 through the MAPK pathway. In conclusion, statins might be potential candidates for TNBC therapy reducing ets-1 expression via overexpression of dusp4.

Triple-negative breast cancer (TNBC) is defined by a lack of expression of estrogen receptor (ER), progesterone receptor (PgR) and overexpression of human epidermal growth factor receptor-2 (HER-2). This cancer accounts for approximately 15–20% of all breast cancers (BCs)^1^,^2^. Although many advances have been made recently in BC treatment, TNBC represents a significant challenge since it is associated with dismal prognosis and no effective targeted therapy has yet been identified. Cytotoxic chemotherapy is currently the only treatment option for TNBC; thus, there is an urgent unmet need to improve treatment outcomes^3^,^4^.

Many studies have endeavored to determine the molecular mechanisms underlying the aggressiveness of TNBC. This aggressiveness was recently found to be associated with expression of dual specificity phosphatase 4 (*DUSP4*), an extracellular-regulated kinase (*ERK*) phosphatase known to be a negative regulator of ERK. Specifically, decreased dusp4 expression resulted in a loss of negative feedback to the Ras-ERK pathway, which facilitated tumor cell proliferation and survival in TNBCs^5^. Moreover, patients with basal-like BC who received neoadjuvant chemotherapy and had low levels of dusp4 expression achieved poorer pathologic complete remission rates and shorter recurrence-free survival periods compared with patients with high levels of dusp4 expression.

The ETS family of transcription factors regulates the expression of genes involved in normal cell development, proliferation, and differentiation^6^. Therefore, the dysregulation of these transcription factors facilitates cell proliferation in many cancers, and several ETS members have been shown to participate in invasion and metastasis by activating gene transcription^7^. V-ets avian erythroblastosis virus E26 oncogene homology 1(*ETS-1*) is the founding member of the ETS family and regulates transcription and DNA binding by binding to ETS-domain proteins^8^,^9^. Previous have demonstrated that *ETS1* is required for activation of the RAS/ERK pathway and migration of RAS*/*ERK-activated cells by transcriptional activation through ETS*/*AP-1 sites^10^,^11^. In invasive BC, upregulation of *ETS1* is associated with high aggressiveness and poor prognosis. Consistent with this observation, *ETS1* regulates the expression of important angiogenetic and extracellular matrix remodeling factors such as *VEGF, MMP2*, and *MMP9*^12^,^13^,^14^,^15^. Furthermore, *ETS1* also represses genes such as *DUSP4, DUSP6*, and *SPRY4*, all of which provide negative feedback to the RAS/ERK pathway^16^,^17^,^18^.

Statins, small molecule inhibitors of 3-hydroxy-3-methylglutaryl-coenzyme A reductase (HMG-CoAR), exert antitumor effects by altering the RAS/MEK/ERK and RAS/PIK3CA/AKT signaling pathways^19^,^20^. Lipophilic statins inhibit the growth and proliferation of BC cells, especially hormone receptor-negative, basal-like BC cells^21^,^22^,^23^.

Based on these studies, we hypothesized that statins suppress TNBC growth by altering the expression of *DUSP4* and *ETS1*.

## Results

### Expression of *DUSP4* and *ETS* family genes in 22 BC cell lines using Western blot, RT-PCR, and nCounter expression assay

**Figure 1:**
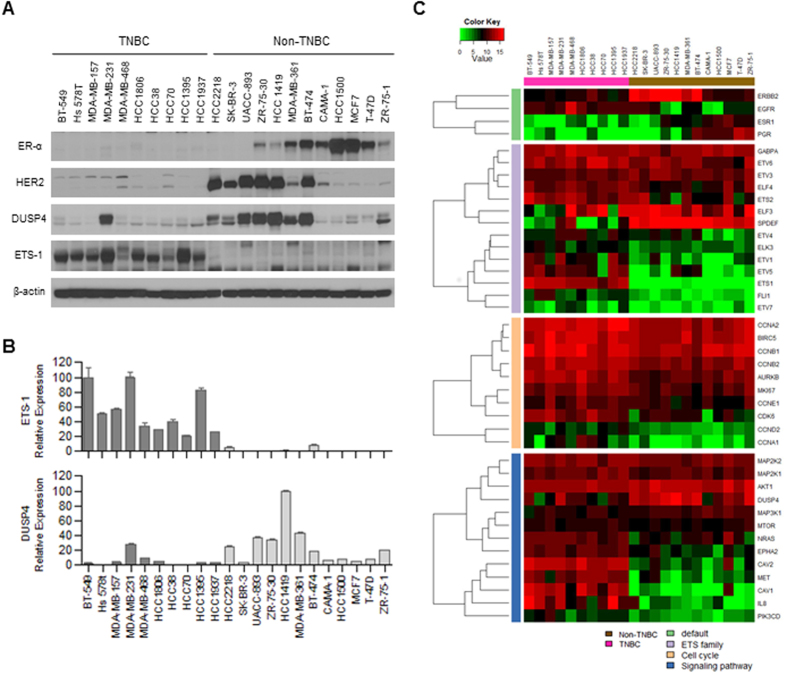
Gene expression characterization of breast cancer cell lines. (**A**) Western blot analysis of BC cell lines with anti-ER-α, anti-HER-2, anti-DUSP4, and anti-ETS1 antibodies. Equal amounts of protein were analyzed using SDS–PAGE; β-actin was included as a loading control. (**B**) Quantitative real-time RT–PCR analysis of the mRNA expression levels of ETS-1 and DUSP4. Columns, means of three experiments; bars, SEMs. (**C**) nCounter expression assay of 70 genes in the BC cell lines.

To compare the levels of dusp4 and ets-1 expression in different BC subtypes, we performed Western blot and RT-PCR analyses of ER, PgR, and HER-2 expression in 22 BC cell lines representing various BC subtypes (Fig. 1A,B). These results were used to classify each of the 22 BC cell lines into TNBC or non-TNBC subtypes.

Western blot analysis showed that dusp4 expression was downregulated in TNBC cell lines compared with non-TNBC cell lines; the opposite effect was observed for ets-1 (Fig. 1A).

We also examined the mRNA expression levels of *ETS1*and *DUSP4* in the 22 BC cell lines using quantitative RT–PCR. The relative *ETS1* mRNA expression levels are shown in Fig. 1B. Consistent with the Western blot analysis, RT-PCR revealed that ets-1 mRNA expression was higher in most TNBC cell lines than in the non-TNBC cell lines. Similarly, DUSP4 expression was decreased in TNBC cell lines compared with non-TNBC cell lines.

To gain a more comprehensive picture of the differences between TNBC versus non-TNBC cells, we next analyzed the expression of 70 additional genes in the 22 BC cell lines (Supplementary Table 1). Of the 27 ETS genes analyzed, *ETS1, ETS2, ETV1, ETV5, ELK3*, and *FLI1* were overexpressed in TNBC cell lines [TNBC vs. non-TNBC; false discovery rate (FDR) = <0.001, 0.014, 0.003, 0.002, <0.001, and <0.001, respectively] (Table 1 and Fig. 1C). In contrast, *SPDEF* genes were expressed at lower levels in TNBC cell lines than in non-TNBC cell lines (FDR = <0.001). We also observed reduced *DUSP4* expression in TNBC cell lines (FDR = 0.008).

Of the 18 cell cycle-related genes examined, *CCNA1, CCND2,* and *CDK6* exhibited significantly higher expression in the TNBC cell lines. Generally, cell cycle-related genes were upregulated in TNBC cell lines.

### Antitumor effects of simvastatin on BC cells *in vitro*

**Figure 2:**
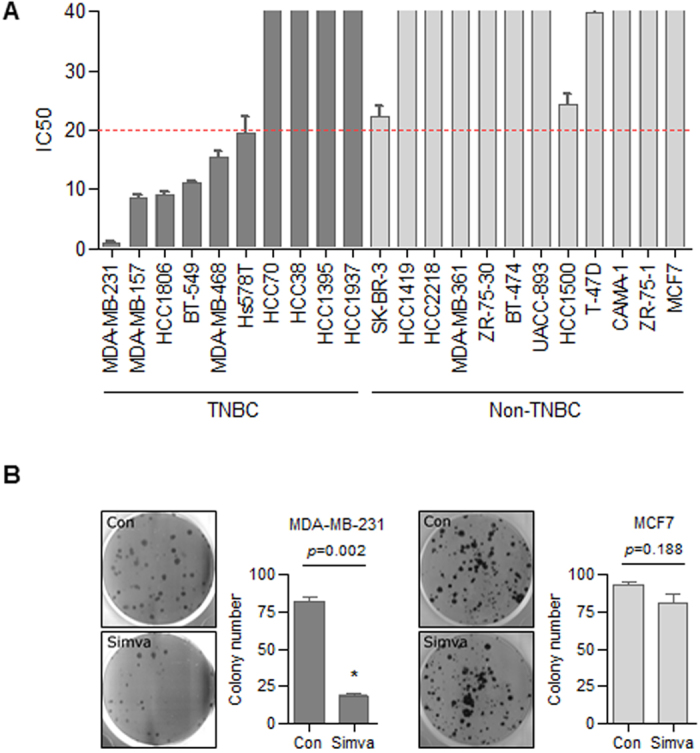
Survival, colony-forming, and cell invasion assays. (**A**) IC_50_ values for a panel of BC cell lines treated with simvastatin. Proliferation was assessed 3 days after treating BC cell lines with varying concentrations (0–120 μmol/L) of simvastatin. Dose-response curves were used to calculate the IC_50_ values. Values are shown are means of three independent experiments. IC_50_ values less than 20 μmol/L (denoted by the red dashed line) indicate a potential therapeutic effect of simvastatin. White columns, TNBC cells; gray columns, HER-2-positive cells; black columns, hormone receptor-positive cells. (**B**) Colony-forming assays of TNBC (MDA-MB-231) and ER-α-expressing (MCF 7) cells. Cells were treated with 0.1 μM simvastatin for 3 days and maintained at 37 °C for 14 days. Fresh medium was added after 7 days. Cells were stained with 0.1% crystal violet.

To determine whether statin inhibits cell proliferation, we performed MTT assays. We found that TNBC cells were sensitized to simvastatin compared with non-TNBC cell lines, as indicated by the IC_50_ values. Six of the 10 TNBC lines tested had an IC_50_ <20 μmol/L, whereas all non-TNBC cell lines had an IC_50_ ≥ 20 μmol/L (Fig. 2A). A colony-forming assay was performed by seeding equal numbers of MDA-MB-231 cells and MCF-7 cells in triplicate on soft agar in 6-well tissue culture plates. After treating the cells with 0.1 μM simvastatin, the MDA-MB-231 cells formed fewer colonies than the MCF-7 cells (Fig. 2B). Thus, stimulation with 0.1 μM simvastatin significantly inhibited colony formation in MDA-MB-231 cells (*P* = 0.002).

### Effects of statin treatment on gene expression in TNBC cell lines

We next sought to determine the effect of simvastatin on the MAP kinase signaling pathway. To this end, we performed a PCR array of 45 genes associated with the MAP kinase signaling pathway using HCC1806 and MDA-MB-231 cells (Table 2). This analysis showed that simvastatin significantly suppressed ETS-1 expression in TNBC cell lines. In addition, we found that the mRNA expression levels of 13 cell cycle-related genes and of the MAP kinase genes *MAP2K3* and *MAP2K6* were also downregulated by statin treatment.

**Figure 3:**
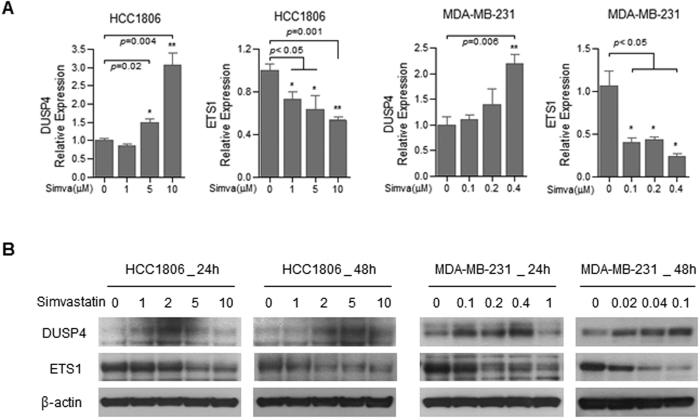
Effects of statin treatment on gene expression. (**A**) Quantitative real-time RT–PCR analysis of the mRNA expression levels of ETS-1 and DUSP4. (**B**) Western blot analysis of TNBC cell lines before and after simvastatin treatment. Blots were probed with anti-DUSP4 and anti-ETS1 antibodies.

In addition, we evaluated the effects of statins on dusp4 and ets-1 expression using RT-PCR and Western blot analyses (Fig. 3A,B). In HCC1806 cells, simvastatin treatment (>5 μM) resulted in decreased ets-1 mRNA expression and increased dusp4 expression (Fig. 3A). MDA-MD-231 cells were more sensitive to simvastatin than HCC1806 cells; 0.4 μM simvastatin was sufficient to affect ets-1 and dusp4 mRNA expression. Western blot analysis also revealed that the effects of simvastatin on ets-1 and dusp4 expression were dose-dependent (Fig. 3B).

Additionally, we evaluated the effects of statin on the gene expression profile in TNBC cells using the nCounter expression assay. Statin treatment restored dusp4 and suppressed ets-1 expression to levels that were not significantly different from those in non-TNBC cells (both *P* = 0.068) (Supplementary Fig. 1).

### Effect of *ETS1* and *DUSP4* expression on the viability of TNBC cells

**Figure 4:**
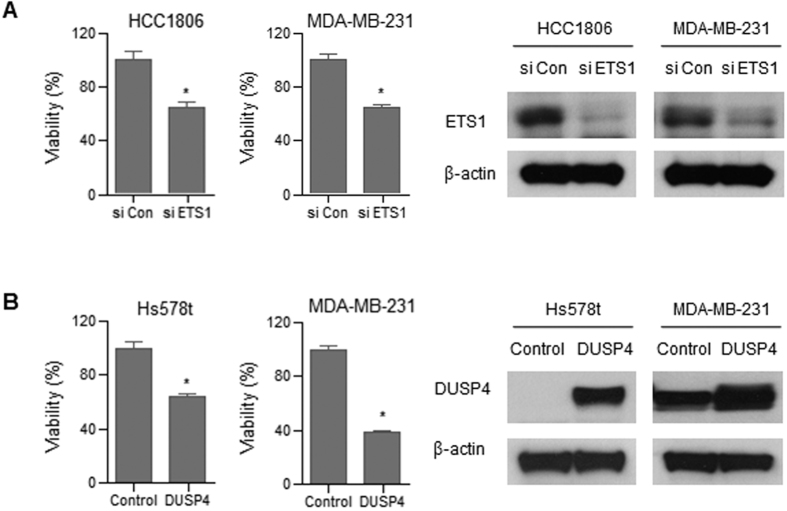
Effects of simvastatin treatment on signaling and transduction pathways. (**A**) TNBC cells were transfected with control siRNA or ETS-1 siRNA for 72 h. Cell viability was determined using the MTT assay. (**B**) TNBC cells were transfected with control vector or a DUSP4 expression vector for 72 h. Cell viability was determined using the MTT assay.

To determine the effects of ets-1 and dusp4 expression on cell viability, we transfected TNBC cells with *ETS1* siRNA. In MTT assay, after transfecting si-ETS1 into HCC1806 and MDA-MB-231 cells, we found that cell viability of both HCC1806 and MDA-MB-231 cells were reduced (*P* < 0.05) (Fig. 4A). We also performed *DUSP4* transfection into TNBC cells. We also analyzed cell viability and ets-1 expression after transfection of Hs578t and MDA-MB-231 cells. Transfection of *DUSP4* resulted in decreased ets-1 expression in TNBC cells. Moreover, ectopic expression of *DUSP4* in the Hs 579t and MDA-MB-231 TNBC cell lines resulted in significantly decreased cell viability compared with empty vector (pcDNA) transfection (*P* < 0.05) (Fig. 4B).

### Inhibition of dusp4 expression blunts simvastatin-induced apoptosis

**Figure 5:**
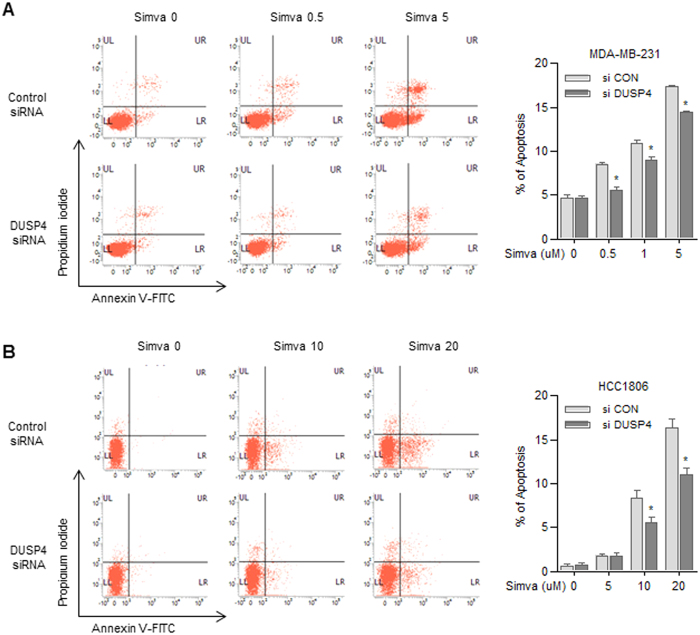
Apoptosis assay. MDA-MB-231 (**A**) and HCC1806 (**B**) cells were transfected with control siRNA or DUSP4-targeting siRNA. At 24 h post-transfection, simvastatin was added and cells were incubated an additional 48 h. The distribution of annexin V-FITC/PI staining is shown. Data are expressed as averages for each time point and were calculated using the results from three independent experiments.

To investigate the mechanism of simvastatin-induced apoptosis, we transfected HCC1806 and MDA-MB-231 cells with *DUSP4*-targeting siRNA and analyzed apoptosis by FACS (Fig. 5). As expected, simvastatin induced apoptosis in cells transfected with control siRNA. In contrast, cells transfected with *DUSP4* siRNA exhibited decreased simvastatin-induced apoptosis. In MDA-MB-231 cells, *DUSP4* siRNA transfection inhibited apoptosis in the presence of 0.5, 1, and 5 μM simvastatin (P < 0.05), whereas *DUSP4* siRNA transfection suppressed apoptosis in HCC1806 cells induced by 1 and 5 μM simvastatin (P < 0.05).

### Dusp4 and ets-1 expression in TNBC cells after *in vitro* treatment with simvastatin, a MEK inhibitor, and a PIK3CA inhibitor

To investigate the downstream signaling pathways activated by simvastatin, the expression levels of mitogen-activated protein kinase (MEK) and phosphatidylinositol 3-kinase (PI3K) were evaluated after simvastatin treatment. TNBC cells were treated with 0, 1, 5, or 10 μM simvastatin, and the expression levels of total and phosphorylated Erk and Akt were assessed by Western blot analysis (Supplementary Fig. 2A). Simvastatin treatment decreased the levels of both phosphorylated Erk and Akt in a dose-dependent manner. We next used the specific pharmacological inhibitors U0126 and LY294002 to inhibit the MEK-Erk and PI3K-Akt pathways, respectively (Supplementary Fig. 2B). U0126-mediated inhibition of MEK abrogated ets-1 overexpression and suppressed Erk phosphorylation in a dose-dependent manner. In a similar pattern, LY294002-mediated PI3K/Akt inhibition decreased ets-1 expression in a dose-dependent manner.

Finally, we performed MTT assays to determine the effect of statin treatment in combination with LY294002 on the viability of BT-549, Hs578t, MDA-MB-231, and MCC 1806 cells. Treatment with either simvastatin or LY294002 alone each resulted in decreased cell viability; moreover, simvastatin and LY294002 acted synergistically to inhibit cell proliferation when given together (Supplementary Fig. 2C).

## Discussion

Previous studies have shown that ETS family members are significantly upregulated in TNBC cells compared with other cells, whereas *DUSP4* is downregulated in TNBC cells^5^,^24^. Our results indicate that statins suppress proliferation of TNBC cell lines by restoring dusp4 expression and suppressing ets-1 upregulation; thus, statins are potential candidate molecules for treating TNBCs.

Our research has several clinical implications for the treatment of TNBCs. *ETS1* might have a role in TNBC pathogenesis, especially for phenotypes resistant to conventional treatment, and could be a biomarker for TNBC cells. Increased ets-1 expression is associated with aberrant transcription of multiple cancer-associated genes, which can result in enhanced energy metabolism, cell survival, matrix degradation, cell growth, angiogenesis, migration, and invasion^13^,^25^. *ETS1* contains a Ras-responsive phosphorylation site at threonine-38, phosphorylation of which strongly increases the transcriptional activity of ets-1. In a complex signal transduction network, ets-1 is an effector of oncogenic Ras via the MAPK pathway^26^,^27^, a main mediator of Ras signaling that is dysregulated in many cancers, particularly BC. In addition, loss of DUSP4 also promotes MARK pathway activation and induces the cancer stem cell-like phenotype of TNBC^24^. Accordingly, the negative correlation of ets-1 and dusp4 expression in TNBC could plausibly contribute to the aggressive nature of TNBC. In this study, we found that *ETS1* and other ETS family members such as *ETS2, ETV1, ETV5, ELK3*, and *FLI1* were overexpressed in TNBC cell lines compared with other BC cell lines. Therefore, the elevated expression of ETS family members (e.g., *ETS1*) might contribute to chemotherapy resistance as well as tumor invasion and aggressiveness in TNBC cells^28^,^29^,^30^.

Our results imply that statins, which are small molecule inhibitors of HMG-CoAR, might be able to potently inhibit TNBC growth by restoring dusp4 expression and downregulating ets-1 expression. Dusp4 expression is consistently lost in TNBC cells^31^, which has been reported to promote resistance to treatment^5^. In addition, statin treatment has been shown to alter Dusp4 expression^32^. We previously reported that simvastatin is a promising candidate for treating TNBCs, especially those with wild-type pten expression^33^. In the present study, we used a PCR array and an nCounter expression assay to demonstrate that dusp4 expression is lost in TNBC cells. We propose that statin effectively suppresses ets-1 expression by restoring dusp4 levels in TNBC cells.

Finally, our results indicate that ets-1 overexpression and dusp4 deficiency, a combination that might contribute to treatment failure in refractory TNBCs, can be overcome by statin treatment with mitogen-activated protein kinase (MEK) inhibitors. Since ETS family members can affect the MAPK pathway^28^, MEKs are potential therapeutic targets for treating TNBC.

In summary, our results suggest that the loss of dusp4, a potential biomarker of treatment-resistant TNBC, is associated with ets-1 overexpression via the PI3K and MAPK pathways. Statin, a small inhibitor of HMG-CoAR, is a potential therapeutic candidate for treatment-resistant TNBC because it can reverse ets-1 overexpression by restoring dusp4 expression.

## Materials and Methods

### Human TNBC cell lines and culture

Twenty-two BC cell lines were purchased from the American Type Culture Collection (Manassas, VA, USA). Each cell line was phenotypically characterized as either TNBC or non-TNBC. BT-549, Hs578T, MDA-MB-157, MDA-MB-231, MDA-MB-468, HCC1806, HCC38, HCC70, HCC1395, and HCC1937 were TNBC cell lines, whereas SK-BR-3, UACC-893, ZR-75-30, HCC1419, MDA-MB-361, BT-474, CAMA-1, HCC1500, MCF-7, T-47D, and ZR-75-1 were non-TNBC cell lines. All cells were grown in RPMI-1640 medium supplemented with 10% fetal bovine serum (FBS), 100 mU/mL penicillin, and 100 μg/mL streptomycin under 5% CO_2_ in humidified air at 37 °C. All media and supplements were purchased from Invitrogen Corp. (Carlsbad, CA, USA).

### Antibodies and reagents

Anti-ER antibodies were purchased from Santa Cruz Biotechnology (Santa Cruz, CA, USA). Anti-ets-1 antibodies were purchased from Abcam (Cambridge, UK). Anti-phospho-ERK1/2 (Thr202/Thr204), anti-ERK1/2, anti-Akt (Ser473), and anti-Akt antibodies were purchased from Cell Signaling Technology (Beverly, MA, USA). Anti-β-actin antibodies and simvastatin were purchased from Sigma-Aldrich (St. Louis, MO, USA); U0126 and LY294002 were from Cell Signaling Technology.

### nCounter expression assay (NanoString)

The expression levels of 70 genes previously reported as belonging to the ETS family or associated with the ETS family (Table 1) were tested in 22 cell lines using the NanoString nCounter Analysis System (NanoString Technologies, Seattle, WA, USA)^34^.

The effects of simvastatin treatment (10 μmol, 24 hr) on the expression profiles of these 70 genes in TNBC cell lines were also investigated using the nCounter expression assay (NanoString).

NanoString nCounter gene expression data were filtered using quality control (QC) criteria according to the manufacturer’s recommendations. Raw counts of QC-passed samples were normalized using five reference genes as internal controls (GUSB, PUM1, TBP, TFRC, and TUBB). All QC and normalization procedures were performed using nSolver Analysis Software v2.0 (NanoString Technologies); all data were log_2_-transformed before further analysis. The student’s *t*-test was used to compare normalized expression values between TNBC and non-TNBC cell lines. P-values were adjusted using the false discovery rate (FDR) method for multiple comparisons^35^. Genes with FDRs below 0.001 and absolute log_2_ fold change greater than 2 were considered to be differentially expressed and are displayed on the volcano plot. Among the genes related to the ETS family, the cell cycle, and signaling pathways, a hierarchically clustered heat map was generated using genes whose adjusted p-values were less than 0.1. The paired *t*-test was applied to identify genes that were differentially expressed before versus after simvastatin treatment. Differential expression was defined as a p-value less than 0.05 and an absolute log_2_ fold change greater than 1.

Intrinsic subtype classification was performed using the PAM50 predictor, as described in Parker *et al*.^36^. R version 3.0.2 (http://www.R-project.org/) was used to perform all statistical tests, generate all plots, and predict all PAM50 subtypes.

### Isolation of RNA and reverse transcription polymerase chain reaction (RT–PCR)

Total cellular RNA was isolated using Trizol (GIBCO BRL/Life Technologies, Carlsbad, CA, USA). For RT–PCR, 2 μg of RNA was treated with RNase-free DNase and cDNA was synthesized with the Omniscript Reverse Transcription kit (QIAGEN). The levels of DUSP4, ets-1, and glyceraldehyde 3-phosphate dehydrogenase (GAPDH; loading control) cDNA were quantified with an ABI Prism 7900 Real-Time PCR system (Applied Biosystems, Foster City, CA, USA). All primers and probes (GAPDH cat. no. Hs99999905-m1; DUSP4 cat. no. Hs01027785; ets-1 cat. no. Hs00428293) were obtained commercially and are proprietary; thus, sequences are not available (TaqMan Gene Expression Assay, Applied Biosystems). The following amplification conditions were used: 50 °C for 2 min and 95 °C for 10 min, followed by 40 cycles of 94 °C for 15 sec and 60 °C for 1 min. Data were analyzed with ABI Prism 7900 SDS 2.3 software (Applied Biosystems).

### Western blot analysis

For Western blot analysis, cells were lysed with RIPA buffer (0.5% sodium deoxycholate, 1% Nonidet P-40, 150 mM NaCl, 50 mM Tris pH 7.5, 0.1% sodium dodecyl sulfate [SDS], and 1 mM PMSF). The protein concentrations of the supernatants were determined using the BCA protein assay (Pierce, Rockford, IL, USA). Equal amounts of protein were loaded onto SDS–polyacrylamide gels and transferred to nitrocellulose membranes. Nonspecific binding sites were blocked by incubating the membranes in blocking solution containing 5% nonfat dry milk for 2 h, after which membranes were incubated with primary antibodies. After incubation with horseradish peroxidase (HRP)-conjugated secondary antibodies, immunoreactive bands were visualized using enhanced chemiluminescent (ECL) reagents (Amersham). To confirm equal protein loading, blots were stripped and reprobed with anti-beta-actin antibodies.

### Cell proliferation and colony formation assays

The effect of simvastatin on BC cell growth *in vitro* was determined by measuring the absorbance of 3-(4,5-dimethylthiazol-2-yl)-2,5-diphenyltetrazolium bromide (MTT) in living cells. Briefly, cells were seeded (3 × 10^3^ cells per well) on 96-well microtiter plates (Nunc, Roskilde, Denmark). After 72 h of drug exposure, 5 mg/mL MTT (Sigma-Aldrich) solution was added to the culture medium (10 μL per 100 μL of medium), and the plates were incubated for an additional 4 h at 37 °C. Excess MTT solution was aspirated. Formazan crystals in viable cells were dissolved in 100 μL of acid-isopropanol (0.04 N HCl in isopropanol), and the absorbance at 540 nM was measured. The half-maximal inhibitory concentration (IC_50_) values were estimated using Prism software (Version 4.0, GraphPad Software Inc., San Diego, CA, USA).

For colony formation assays, cells were plated in 6-well plates (Nunc; 2 × 10^2^ cells/well for MCF7 cells and 1 × 10^2^ cells/well for MDA-MB-231 cells) and treated with 0.1 μM simvastatin for 3 days. Triplicate cultures of each cell type were maintained at 37 °C for 14 days under 5% CO_2_ in humidified air. Fresh medium was added after 7 days, and cells were stained with 0.1% crystal violet. Colonies, defined as groups of at least 50 cells, were counted under an inverted phase contrast microscope (CK × 31SF; Olympus Biosystems, Hamburg, Germany). The percentage of relative cell proliferation is expressed as (number of colonies from treated cells/number of colonies from controls) × 100. Each assay was repeated three times.

### Ectopic expression of DUSP4 in TNBC cell lines

The DUSP4-pCMV6-Entry expression plasmid was purchased from Origene. Cells were transfected with 4 *μ*g of the DUSP4-pCMV6-Entry plasmid using the Neon Transfection System (Invitrogen). Briefly, cells (2 × 10^6^ per transfection) were harvested, washed with PBS, resuspended in 100 *μl* of resuspension buffer, and electroporated according to the manufacturer’s instructions.

### siRNA transfection

TNBC cells were transfected with siGENOME SMARTpool silencing (si)RNA targeting human *ETS1* or *DUSP4*. Alternatively, cells were transfected with control siRNA. All siRNAs were purchased from Dharmacon (Lafayette, CO, USA). Transfections were performed using the Neon Transfection System according to the manufacturer’s instructions.

### Apoptosis assay

TNBC cells were transfected with DUSP4 siRNA. At 24 h post-transfection, cells were treated with simvastatin for an additional 48 h. For FACS analysis of cell death, cells were stained with Annexin V-FITC (BD Biosciences) and propidium iodide (PI, Sigma) following the manufacturer’s protocol. Briefly, 100 μl of cell suspension (1 × 10^5^ cells in 1X binding buffer) was stained with 2.5 μl of Annexin V-FITC and 5 μl of PI [50 μg/ml]. The suspension was gently mixed and incubated for 15 min at room temperature in the dark, after which 200 μl of 1X Annexin binding buffer was added. Cells were immediately analyzed on a FACS VERSE flow cytometer. Data from 10,000 cells were collected at a low flow rate and analyzed using BD FACSuite (BD Biosciences).

### Statistical analysis

Comparisons between experimental groups and the relevant controls were performed using the student’s *t*-test. P values < 0.05 were considered to indicate significant differences. All experiments were performed at least three times.

## Additional Information

**How to cite this article**: Jung, H. H. *et al*. Statins affect *ETS1*-overexpressing triple-negative breast cancer cells by restoring *DUSP4* deficiency. *Sci. Rep.* **6**, 33035; doi: 10.1038/srep33035 (2016).

## Supplementary Material

Supplementary Information

## Figures and Tables

**Table 1:** Seventy gene expression profiles of 22 breast cancer cell lines.

Gene	Non-TNBC	TNBC	P-value	FDR	Gene	Non-TNBC	TNBC	P-value	FDR
EGFR	6.06	10.71	1.56.E-03	6.54.E-03	CCNA2	10.59	11.88	4.55.E-04	2.64.E-03
ERBB2	13.20	8.97	3.92.E-04	2.33.E-03	CCNB1	11.08	12.00	9.11.E-03	2.78.E-02
ESR1	7.30	2.47	1.53.E-03	6.54.E-03	CCNB2	9.83	10.78	3.96.E-03	1.34.E-02
PGR	7.04	1.50	6.46.E-03	2.07.E-02	CCND1	11.33	10.44	7.76.E-02	1.42.E-01
EHF	8.80	6.64	2.46.E-01	3.56.E-01	CCND2	3.17	5.55	1.16.E-04	9.53.E-04
ELF1	9.57	9.47	7.97.E-01	8.66.E-01	CCNE1	7.61	8.73	1.24.E-02	3.57.E-02
ELF2	10.34	10.21	5.72.E-01	7.22.E-01	CDK4	10.41	10.68	2.08.E-01	3.21.E-01
ELF3	10.40	7.51	4.68.E-02	9.67.E-02	CDK6	6.44	9.57	3.28.E-04	2.28.E-03
ELF4	7.52	8.77	2.58.E-03	9.63.E-03	CDKN1A	8.43	7.96	4.69.E-01	6.11.E-01
ELF5	4.49	2.37	1.53.E-01	2.46.E-01	CDKN2A	2.74	4.94	1.62.E-01	2.58.E-01
ELK1	7.38	7.61	4.68.E-01	6.11.E-01	E2F1	8.85	9.01	5.96.E-01	7.30.E-01
ELK3	3.83	5.93	1.04.E-04	9.53.E-04	MCL1	11.73	11.70	9.13.E-01	9.40.E-01
ELK4	0.82	0.49	6.09.E-01	7.33.E-01	MKI67	8.59	9.68	5.13.E-03	1.69.E-02
ERF	8.20	8.08	7.07.E-01	7.97.E-01	MYC	10.07	10.75	2.31.E-01	3.42.E-01
ERG	0.58	0.75	8.21.E-01	8.71.E-01	AKT1	11.92	11.18	1.28.E-03	5.82.E-03
ETS1	0.27	9.99	5.54.E-12	1.39.E-09	AKT2	9.98	9.70	2.97.E-01	4.18.E-01
ETS2	7.06	9.40	4.09.E-03	1.36.E-02	CAV1	1.78	10.89	1.43.E-08	1.19.E-06
ETV1	2.83	6.96	5.00.E-04	2.84.E-03	CAV2	5.14	12.00	6.10.E-07	3.05.E-05
ETV2	4.66	4.48	5.90.E-01	7.27.E-01	DUSP4	11.38	8.08	1.97.E-03	7.57.E-03
ETV3	8.74	8.09	2.07.E-02	5.23.E-02	EPHA2	6.37	9.59	3.74.E-05	5.19.E-04
ETV4	4.65	6.44	3.53.E-02	7.95.E-02	IL8	3.09	10.01	1.27.E-05	2.45.E-04
ETV5	2.58	7.85	1.38.E-04	1.08.E-03	INPP4B	9.30	8.34	1.20.E-01	2.03.E-01
ETV6	8.85	9.87	3.09.E-02	7.15.E-02	MAP2K1	9.33	10.06	2.38.E-02	5.67.E-02
ETV7	1.06	2.83	4.20.E-02	8.90.E-02	MAP2K2	10.10	10.60	3.60.E-02	7.98.E-02
FEV	1.02	1.49	5.44.E-01	6.91.E-01	MAP2K4	8.71	9.11	9.81.E-02	1.70.E-01
FLI1	1.45	5.28	1.11.E-05	2.32.E-04	MAP3K1	9.02	7.93	1.04.E-02	3.05.E-02
GABPA	9.54	10.00	4.68.E-02	9.67.E-02	MAPK1	11.02	11.35	2.32.E-01	3.42.E-01
SPDEF	11.62	3.95	5.22.E-05	6.21.E-04	MAPK3	10.19	9.47	7.66.E-02	1.41.E-01
SPI1	0.05	0.94	5.50.E-02	1.10.E-01	MET	4.09	9.95	7.75.E-06	1.94.E-04
SPIB	0.34	0.33	9.89.E-01	9.89.E-01	MTOR	7.69	8.28	2.13.E-02	5.23.E-02
SPIC	0.80	0.98	7.26.E-01	8.05.E-01	NRAS	7.42	9.56	2.64.E-03	9.71.E-03
AURKA	10.31	10.69	3.63.E-01	4.96.E-01	PIK3CA	8.59	8.91	6.26.E-02	1.19.E-01
AURKB	9.03	10.75	4.16.E-05	5.47.E-04	PIK3CD	3.09	6.18	4.73.E-05	5.92.E-04
BIRC5	10.71	11.53	3.61.E-02	7.98.E-02	PIK3R1	8.79	8.13	2.97.E-01	4.18.E-01
CCNA1	1.58	5.76	5.44.E-04	3.02.E-03	PTEN	11.44	9.56	6.22.E-02	1.19.E-01

**Table 2:** Human MAP Kinase Signaling Pathway RT2 Profiler^TM^ PCR Array.

Gene	HCC1806		MDA-MB-231	
	Fold regulation	*p-value*	Fold regulation	*p-value*
ETS1	−1.84	0.00448	−2.76	0.01729
CCNA2	−3.09	*0.00558*	−2.75	*0.00300*
CCNB1	−2.30	*0.00094*	−1.75	*0.00037*
CCNB2	−2.42	*0.01402*	−1.68	*0.00062*
CCND1	−2.63	*0.01799*	−1.70	*0.00144*
CCND3	−2.53	*0.01683*	−1.74	*0.00158*
CCNE1	−2.42	*0.00016*	−3.65	*0.00021*
CDK2	−2.15	*0.00274*	−2.24	*0.00051*
CDK4	−2.82	*0.00206*	−2.35	*0.01359*
CDK6	−1.73	*0.00475*	−1.78	*0.00263*
CDKN1A (p21)	9.06	*0.00087*	2.30	*0.00026*
CDKN1C (p57)	3.12	*0.00036*	3.10	*0.00036*
CDKN2C (p18)	−4.39	*0.00042*	−1.70	*0.00101*
E2F1	−7.94	*0.00019*	−4.81	*0.00118*
MAP2K3	−1.67	*0.02651*	−1.70	*0.00023*
MAP2K6	−18.34	*0.00145*	−1.82	*0.00014*

## References

[b1] Perou C. M. . Molecular portraits of human breast tumours. Nature 406, 747–752 (2000). 10963602 10.1038/35021093

[b2] Kreike B. . Gene expression profiling and histopathological characterization of triple-negative/basal-like breast carcinomas. Breast cancer research: BCR 9, R65 (2007). 17910759 10.1186/bcr1771 PMC2242660

[b3] Hudis C. A. & Gianni L. Triple-negative breast cancer: an unmet medical need. The oncologist 16 Suppl 1, 1–11 (2011). 21278435 10.1634/theoncologist.2011-S1-01

[b4] Bauer K. R., Brown M., Cress R. D., Parise C. A. & Caggiano V. Descriptive analysis of estrogen receptor (ER)-negative, progesterone receptor (PR)-negative, and HER2-negative invasive breast cancer, the so-called triple-negative phenotype: a population-based study from the California cancer Registry. Cancer 109, 1721–1728 (2007). 17387718 10.1002/cncr.22618

[b5] Balko J. M. . Profiling of residual breast cancers after neoadjuvant chemotherapy identifies DUSP4 deficiency as a mechanism of drug resistance. Nature medicine 18, 1052–1059 (2012). 10.1038/nm.2795 PMC3693569 22683778

[b6] Sharrocks A. D. The ETS-domain transcription factor family. Nature reviews Molecular cell biology 2, 827–837 (2001). 11715049 10.1038/35099076

[b7] Oikawa T. & Yamada T. Molecular biology of the Ets family of transcription factors. Gene 303, 11–34 (2003). 12559563 10.1016/s0378-1119(02)01156-3

[b8] Goetz T. L., Gu T. L., Speck N. A. & Graves B. J. Auto-inhibition of Ets-1 is counteracted by DNA binding cooperativity with core-binding factor alpha2. Molecular and cellular biology 20, 81–90 (2000). 10594011 10.1128/mcb.20.1.81-90.2000 PMC85055

[b9] Kim W. Y. . Mutual activation of Ets-1 and AML1 DNA binding by direct interaction of their autoinhibitory domains. The EMBO journal 18, 1609–1620 (1999). 10075931 10.1093/emboj/18.6.1609 PMC1171248

[b10] Chang F. . Signal transduction mediated by the Ras/Raf/MEK/ERK pathway from cytokine receptors to transcription factors: potential targeting for therapeutic intervention. Leukemia 17, 1263–1293 (2003). 12835716 10.1038/sj.leu.2402945

[b11] Hollenhorst P. C., McIntosh L. P. & Graves B. J. Genomic and biochemical insights into the specificity of ETS transcription factors. Annual review of biochemistry 80, 437–471 (2011). 10.1146/annurev.biochem.79.081507.103945 PMC5568663 21548782

[b12] Span P. N. . Expression of the transcription factor Ets-1 is an independent prognostic marker for relapse-free survival in breast cancer. Oncogene 21, 8506–8509 (2002). 12466970 10.1038/sj.onc.1206040

[b13] Furlan A. . Ets-1 controls breast cancer cell balance between invasion and growth. International journal of cancer Journal international du cancer 135, 2317–2328 (2014). 24706481 10.1002/ijc.28881

[b14] Katayama S., Nakayama T., Ito M., Naito S. & Sekine I. Expression of the ets-1 proto-oncogene in human breast carcinoma: differential expression with histological grading and growth pattern. Histology and histopathology 20, 119–126 (2005). 15578430 10.14670/HH-20.119

[b15] Park Y. H., Jung H. H., Ahn J. S. & Im Y. H. Ets-1 upregulates HER2-induced MMP-1 expression in breast cancer cells. Biochemical and biophysical research communications 377, 389–394 (2008). 18851945 10.1016/j.bbrc.2008.09.135

[b16] Hollenhorst P. C. . Oncogenic ETS proteins mimic activated RAS/MAPK signaling in prostate cells. Genes & development 25, 2147–2157 (2011). 22012618 10.1101/gad.17546311 PMC3205585

[b17] Selvaraj N., Budka J. A., Ferris M. W., Jerde T. J. & Hollenhorst P. C. Prostate cancer ETS rearrangements switch a cell migration gene expression program from RAS/ERK to PI3K/AKT regulation. Molecular cancer 13, 61 (2014). 24642271 10.1186/1476-4598-13-61 PMC3999933

[b18] Plotnik J. P., Budka J. A., Ferris M. W. & Hollenhorst P. C. ETS1 is a genome-wide effector of RAS/ERK signaling in epithelial cells. Nucleic acids research 42, 11928–11940 (2014). 25294825 10.1093/nar/gku929 PMC4231772

[b19] Yanae M. . Statin-induced apoptosis via the suppression of ERK1/2 and Akt activation by inhibition of the geranylgeranyl-pyrophosphate biosynthesis in glioblastoma. Journal of experimental & clinical cancer research: CR 30, 74 (2011). 21831290 10.1186/1756-9966-30-74 PMC3163617

[b20] Tsubaki M. . Blockade of the Ras/MEK/ERK and Ras/PI3K/Akt pathways by statins reduces the expression of bFGF, HGF, and TGF-beta as angiogenic factors in mouse osteosarcoma. Cytokine 54, 100–107 (2011). 21292498 10.1016/j.cyto.2011.01.005

[b21] Goard C. A. . Identifying molecular features that distinguish fluvastatin-sensitive breast tumor cells. Breast cancer research and treatment 143, 301–312 (2014). 24337703 10.1007/s10549-013-2800-y

[b22] Campbell M. J. . Breast cancer growth prevention by statins. Cancer research 66, 8707–8714 (2006). 16951186 10.1158/0008-5472.CAN-05-4061

[b23] Garwood E. R. . Fluvastatin reduces proliferation and increases apoptosis in women with high grade breast cancer. Breast cancer research and treatment 119, 137–144 (2010). 19728082 10.1007/s10549-009-0507-x PMC4087110

[b24] Balko J. M. . Activation of MAPK pathways due to DUSP4 loss promotes cancer stem cell-like phenotypes in basal-like breast cancer. Cancer research 73, 6346–6358 (2013). 23966295 10.1158/0008-5472.CAN-13-1385 PMC4090144

[b25] Verschoor M. L., Wilson L. A., Verschoor C. P. & Singh G. Ets-1 regulates energy metabolism in cancer cells. PloS one 5, e13565 (2010). 21042593 10.1371/journal.pone.0013565 PMC2962648

[b26] Yang B. S. . Ras-mediated phosphorylation of a conserved threonine residue enhances the transactivation activities of c-Ets1 and c-Ets2. Molecular and cellular biology 16, 538–547 (1996). 8552081 10.1128/mcb.16.2.538 PMC231032

[b27] Wasylyk C., Bradford A. P., Gutierrez-Hartmann A. & Wasylyk B. Conserved mechanisms of Ras regulation of evolutionary related transcription factors, Ets1 and Pointed P2. Oncogene 14, 899–913 (1997). 9050989 10.1038/sj.onc.1200914

[b28] Wallace J. A. . Ets2 in tumor fibroblasts promotes angiogenesis in breast cancer. PloS one 8, e71533 (2013). 23977064 10.1371/journal.pone.0071533 PMC3745457

[b29] Yuan Z. Y. . Overexpression of ETV4 protein in triple-negative breast cancer is associated with a higher risk of distant metastasis. OncoTargets and therapy 7, 1733–1742 (2014). 25328406 10.2147/OTT.S66692 PMC4196788

[b30] Scheiber M. N. . FLI1 expression is correlated with breast cancer cellular growth, migration, and invasion and altered gene expression. Neoplasia (New York, NY) 16, 801–813 (2014). 10.1016/j.neo.2014.08.007 PMC4212256 25379017

[b31] Manzano R. G., Martinez-Navarro E. M., Forteza J. & Brugarolas A. Microarray phosphatome profiling of breast cancer patients unveils a complex phosphatase regulatory role of the MAPK and PI3K pathways in estrogen receptor-negative breast cancers. International journal of oncology 45, 2250–2266 (2014). 25201346 10.3892/ijo.2014.2648 PMC4215587

[b32] Bjarnadottir O. . Global Transcriptional Changes Following Statin Treatment in Breast Cancer. Clin Cancer Res 21, 3402–3411 (2015). 25840970 10.1158/1078-0432.CCR-14-1403

[b33] Park Y. H., Jung H. H., Ahn J. S. & Im Y. H. Statin induces inhibition of triple negative breast cancer (TNBC) cells via PI3K pathway. Biochemical and biophysical research communications 439, 275–279 (2013). 23973711 10.1016/j.bbrc.2013.08.043

[b34] Park Y. H. . A seven-gene signature can predict distant recurrence in patients with triple-negative breast cancers who receive adjuvant chemotherapy following surgery. International journal of cancer Journal international du cancer 136, 1976–1984 (2015). 25537444 10.1002/ijc.29233

[b35] Benjamini Y. & Hochberg Y. Controlling the False Discovery Rate - a Practical and Powerful Approach to Multiple Testing. J Roy Stat Soc B Met 57, 289–300 (1995).

[b36] Parker J. S. . Supervised risk predictor of breast cancer based on intrinsic subtypes. Journal of clinical oncology: official journal of the American Society of Clinical Oncology 27, 1160–1167 (2009). 19204204 10.1200/JCO.2008.18.1370 PMC2667820

