# Supplementary material for: Statins affect ETS1-overexpressing triple-negative breast cancer cells by restoring DUSP4 deficiency
Source: Sci Rep. 2016 Sep 8;6:33035. doi: 10.1038/srep33035 (PMC5015082; doi:10.1038/srep33035)

Supplementary Data Information

Statins affect *ETS-1*-overexpressing triple-negative breast cancer cells by restoring *DUSP4* deficiency

Hae Hyun Jung[1,4,†] Soohyeon Lee[2,†], Ji-Yeon Kim[2], Jin Seok Ahn[2], Yeon Hee Park[1,2,4, *] & Young-Hyuck Im[1,2,*]

Supplementary Figure 1.Effects of simvastatin treatment on level of gene expression

(**A**) nCounter expression assay of four TNBC cell lines(BT-549, Hs578t, MDA-MB-231 and HCC1806) before and after simvastatin treatment. (B) ETS1, CDK4, CCNE1, CDKN1A, MET, and ETS1 expression changes in BT-549, Hs578t, MDA-MB-231 and HCC1806 cell lines after simvastatin treatment. The simvastatin dose was 10 μmol.

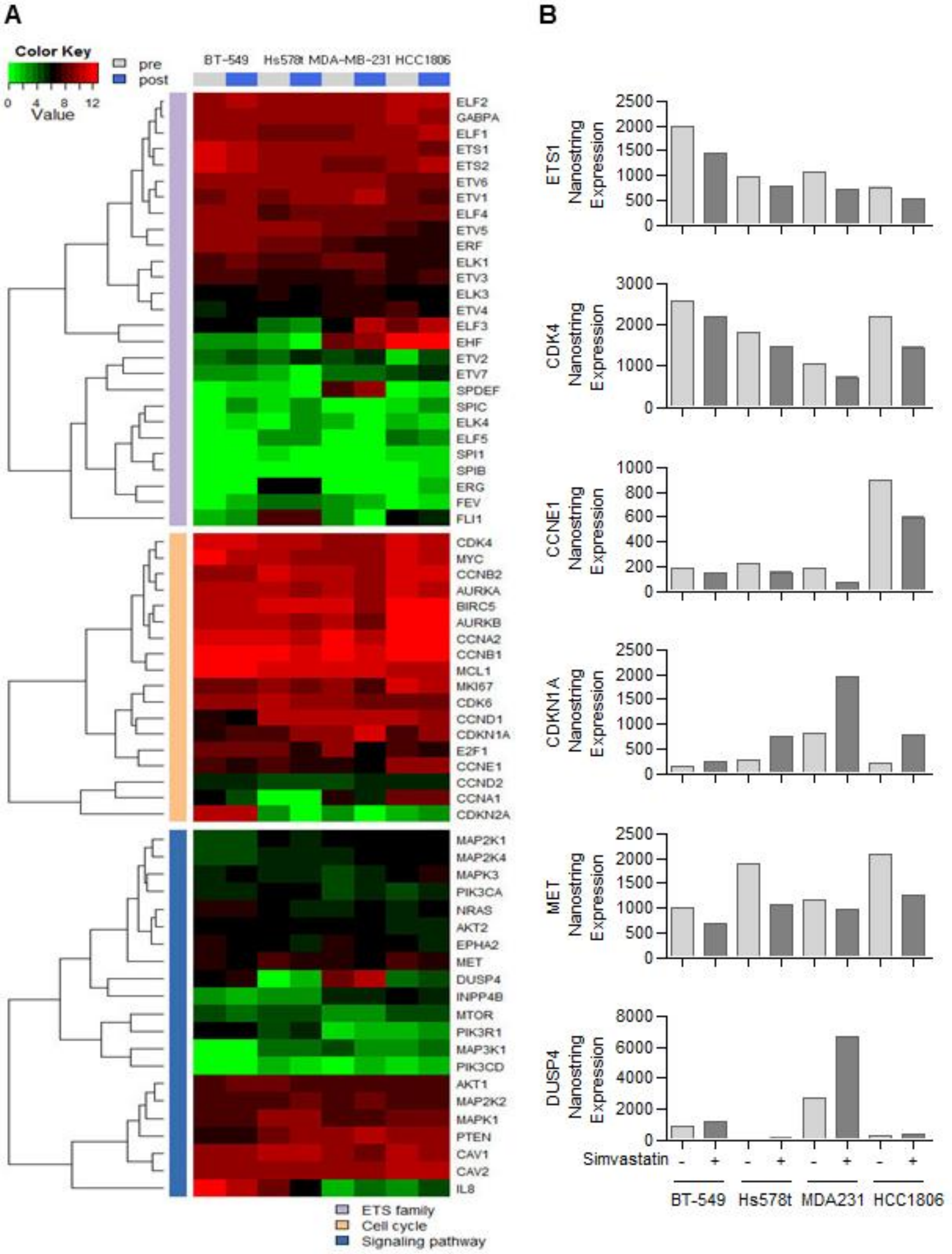

Supplementary Figure 2. Effects of simvastatin treatment on signaling and transduction pathways. (**A**) Effects of simvastatin on ERK and AKT signaling pathways in TNBC cells. Cell (BT549, Hs578t, MDA-MB-231, and HCC1806) were treated with simvastatin at 0, 1, 5, or 10 μM for 24 h. Cell lysates were prepared and analyzed using immunoblotting with anti-phospho ERK1/2 and Akt antibodies as well as anti-total ERK and Akt antibodies; β-actin was used as a loading control. (**B**) The effects of MAPK/AKT pathway inhibitors combined to simvastatin on ets-1 expression. TNBC cell lines (BT549, Hs578t, MDA-MB-231, and HCC1806) were treated with U0126 or LY294002 for 24 h. Cell lysates were prepared and analyzed using immunoblotting with anti-phospho ERK1/2 and Akt antibodies as well as anti-total ERK and Akt antibodies; β-actin was used as a loading control. (**C**) The effects of LY294002 combined to simvastatin on BT-549, Hs578t, MDA-MB-231, HCC1806 cell lines.

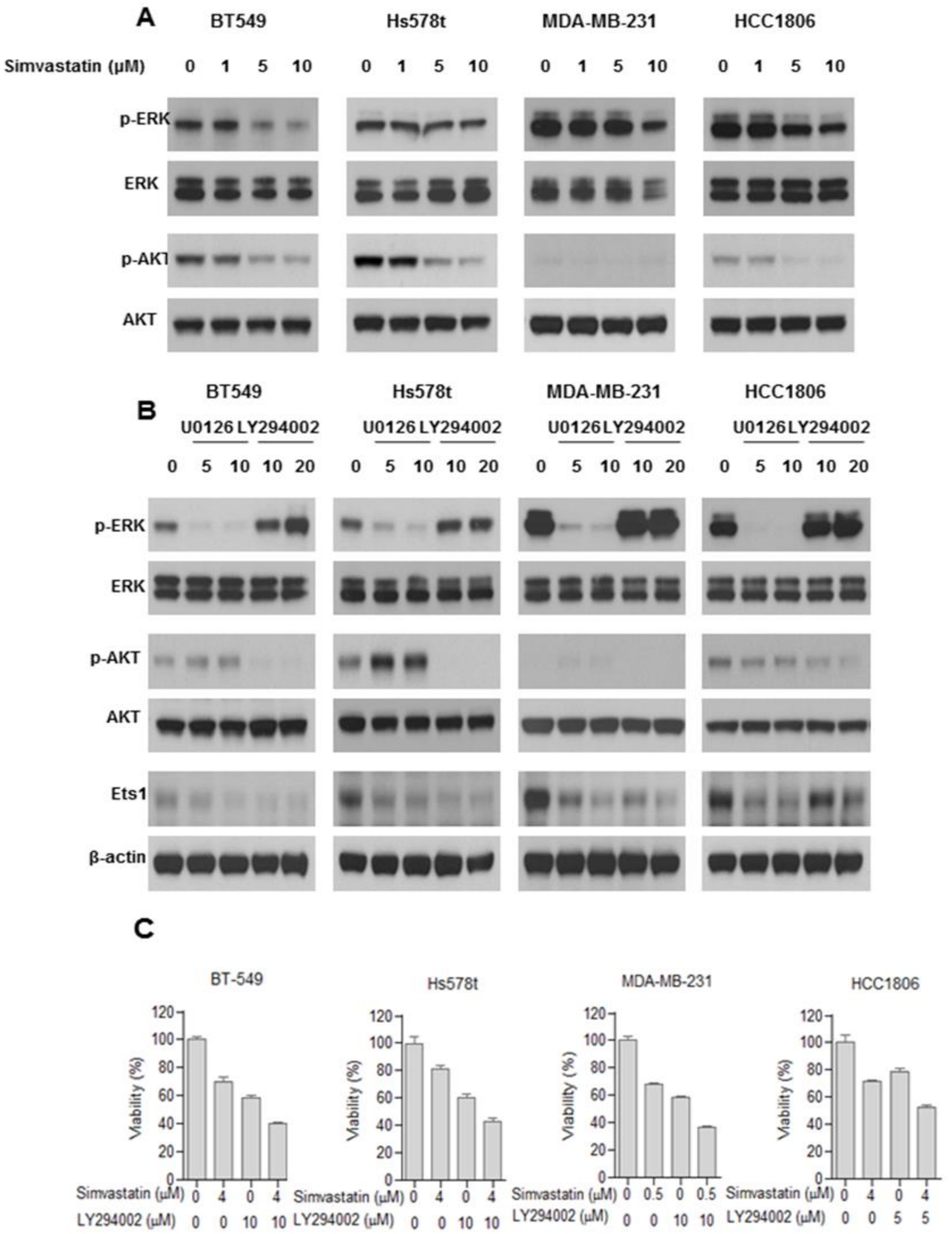

Supplement: Supplementary Information [file srep33035-s1.pdf]
